# Enhancing review criteria for dissemination and implementation science grants

**DOI:** 10.1186/s43058-023-00399-2

**Published:** 2023-02-21

**Authors:** Nicole A. Stadnick, Clare Viglione, Erika L. Crable, Jessica L. Montoya, Maryam Gholami, Irene Su, Borsika Rabin

**Affiliations:** 1 Dissemination and Implementation Science Center, UC San Diego Altman Clinical and Translational Research Institute, La Jolla, USA; 2 Department of Psychiatry, University of California San Diego, La Jolla, USA; 3 Child and Adolescent Services Research Center, San Diego, USA; 4 Herbert Wertheim School of Public Health and Human Longevity Science, University of California San Diego, La Jolla, USA; 5 Department of Obstetrics, Gynecology, and Reproductive Sciences, University of California San Diego, La Jolla, USA

**Keywords:** Review criteria, Grants, Research proposals, Dissemination and implementation science

## Abstract

**Background:**

The existing grant review criteria do not consider unique methods and priorities of Dissemination and Implementation Science (DIS). The ImplemeNtation and Improvement Science Proposals Evaluation CriTeria (INSPECT) scoring system includes 10 criteria based on Proctor et al.’s “ten key ingredients” and was developed to support the assessment of DIS research proposals. We describe how we adapted INSPECT and used it in combination with the NIH scoring system to evaluate pilot DIS study proposals through our DIS Center.

**Methods:**

We adapted INSPECT to broaden considerations for diverse DIS settings and concepts (e.g., explicitly including dissemination and implementation methods). Five PhD-level researchers with intermediate to advanced DIS knowledge were trained to conduct reviews of seven grant applications using both the INSPECT and NIH criteria. The INSPECT overall scores range from 0 to 30 (higher scores are better), and the NIH overall scores range from 1 to 9 (lower scores are better). Each grant was independently reviewed by two reviewers, then discussed in a group meeting to compare the experiences using both criteria to evaluate the proposal and to finalize scoring decisions. A follow-up survey was sent to grant reviewers to solicit further reflections on each scoring criterion.

**Results:**

Averaged across reviewers, the INSPECT overall scores ranged from 13 to 24, while the NIH overall scores ranged from 2 to 5. Reviewer reflections highlighted the unique value and utility for each scoring criterion. The NIH criteria had a broad scientific purview and were better suited to evaluate more effectiveness-focused and pre-implementation proposals not testing implementation strategies. The INSPECT criteria were easier to rate in terms of the quality of integrating DIS considerations into the proposal and to assess the potential for generalizability, real-world feasibility, and impact. Overall, reviewers noted that INSPECT was a helpful tool to guide DIS research proposal writing.

**Conclusions:**

We confirmed complementarity in using both scoring criteria in our pilot study grant proposal review and highlighted the utility of INSPECT as a potential DIS resource for training and capacity building. Possible refinements to INSPECT include more explicit reviewer guidance on assessing pre-implementation proposals, providing reviewers with the opportunity to submit written commentary with each numerical rating, and greater clarity on rating criteria with overlapping descriptions.

**Supplementary Information:**

The online version contains supplementary material available at 10.1186/s43058-023-00399-2.

Contributions to the literature

- Advances the application of specific dissemination and implementation science grant review criteria (INSPECT) that can be used by other funders of implementation research
- Provides specific recommendations for combining existing grant review criteria (i.e., NIH) with implementation of science-specific review criteria (i.e., INSPECT) to promote comprehensive proposal reviews
- Identifies refinements to and broadens the rating criteria and expands it with reviewer training materials and reviewer procedures to enhance the applicability and ease of reviewing dissemination and implementation science grant proposals

## Background

The dissemination and implementation science (DIS) field is unique in its purpose and methods that aim to select and test the strategies that accelerate the uptake, implementation, and sustainment of evidence-based interventions for improved public health [1]. DIS research has increasingly gained attention and interest from researchers, health systems, and funders in recent decades [1, 2]. However, compared to basic science, efficacy, or effectiveness research, DIS research proposals may not readily translate for evaluation within traditional biomedical review frameworks such as the National Institutes of Health (NIH) scoring system [3]. Traditional biomedical review systems emphasize innovation and may inadvertently undervalue the contributions of DIS studies that aim to understand the strategies and mechanisms for advancing our knowledge of the feasibility, fidelity, sustainability, scale, and spread of evidence-based practices, programs, and policies. To facilitate rigorous, and relevant reviews of DIS proposals, there is a need for greater availability of pragmatic DIS proposal evaluation tools [4].

To address the need for proposal review systems that are specific to implementation science proposals, Crable and colleagues [5] developed the ImplemeNtation and Improvement Science Proposals Evaluation CriTeria (INSPECT). The INSPECT scoring system operationalizes 10 recommendations described by Proctor et al. [6] as key ingredients for writing compelling implementation science grant proposals. Each key ingredient was translated into a distinct criterion and standardized on a 4-point scale (0–3) with higher scores indicating that all the requirements were met for a given criterion. A cumulative score across all INSPECT criteria ranges between 0 and 30; scores closer to 30 indicate more favorable proposals. The INSPECT scoring system was originally evaluated for utility and reliability using 30 implementation and improvement science pilot study proposals submitted to a university DIS center. Proposals were evaluated with both the NIH scoring criteria and the newly developed INSPECT. Overall, INSPECT demonstrated high inter-rater reliability for applying the scoring system overall (*α* = 0.88) and for each individual criterion [5].

### Current adaptation and application of INSPECT

The University of California San Diego Altman Clinical and Translational Research Institute Dissemination and Implementation Science Center (ACTRI DISC) was founded in 2020 based on an intramural investment to build capacity for DIS research and practice. The ACTRI DISC developed an annual request for applications for 1-year pilot study proposals for investigators to receive up to $20,000 and distributed the pilot study RFA in September 2020 and September 2021. The goal of these DIS pilot study proposals was to increase the dissemination, adoption, implementation, and sustainment of evidence-based interventions by local healthcare organizations, providers, and systems in San Diego and Imperial Counties. To anchor the review of the pilot study proposals, the INSPECT scoring system and the NIH scoring framework were used to rate each application. The request for applications explained that multiple review criteria would be used.

Several adaptations were required for INSPECT to fit within the ACTRI DISC context and to align with our request for applications. Below is a summary of our INSPECT adaptations. Please see Additional file 1 for the specific adaptations to each criterion.

1. Removed reference to “safety net settings”. The INSPECT developers included scoring requirements that prioritized DIS research being conducted in settings that deliver a significant level of health services to the publicly insured, uninsured, underinsured, and other systematically disadvantaged populations (i.e., “safety net”) due to their DIS center’s presence in an academic medical center serving the safety net population. These requirements are not relevant to the ACTRI DISC and were removed.
2. Removed “improvement science” and referred to DIS studies/methods. The INSPECT developers originally included a dual focus on implementation science and improvement science to encourage research proposals that studies the uptake of evidence into routine practice and the healthcare delivery outcomes associated with evidence use.
3. Replaced “conceptual model and theoretical justification” with “conceptual model, theory, or framework” to increase clarity that models, theories, and/or frameworks were acceptable.
4. Replaced the term “treatment” with the broader term of “intervention” that better reflects the diversity of programs, practices, policies, etc. that a DIS project may address.
5. Replaced broader “stakeholder” language to specify that the types of partners who might be engaged in the DIS project could explicitly include consumers/service recipients, providers, leaders, and policymakers.
6. Aligned INSPECT criteria with the ACTRI DISC request for applications. For example, the ACTRI DISC did not require letters of support (as they were in the original INSPECT study) but allowed for letters to be used as potential review material to satisfy the “setting’s readiness to adopt new program” INPECT criteria.
7. Revised the INSPECT “measurement and analysis section” requirements to include requirements for psychometric quality and pragmatic characteristics of any proposed measures in pilot studies. The adapted INSPECT also de-emphasized the requirement for applicants to provide robust data analytic plans describing how all variables and outcomes would be measured.
8. Provided a space to invite reviewers to offer optional written comments justifying each numerical rating.

The objective of this manuscript is to report a case example of how another DIS university center adapted INSPECT and used it in combination with the NIH scoring system to evaluate pilot DIS study proposals. A comparison in scores between this and the original INSPECT study [5] is reported, along with recommendations for refining INSPECT criteria.

## Methods

A total of five PhD-level researchers from public health, psychiatry, and medicine with intermediate to advanced DIS knowledge were trained to review pilot study applications using the adapted INSPECT and the original NIH criteria. These reviewers were recruited from ACTRI DISC’s community of > 500 members. A condition of free ACTRI DISC membership is participation in ACTRI DISC programs and events, such as serving as a pilot grant reviewer. These particular reviewers were invited from the ACTRI DISC membership because of their breadth of topical expertise and the depth of their DIS experience. One of the reviewers was also the lead INSPECT developer and co-author (ELC). Reviewers participated in an initial 1-h group orientation led by NAS, CV, and BR that included review, discussion, and practice using the INSPECT and NIH scoring systems. In addition to this group orientation, reviewers were provided with a summary of reviewer guidance, written instructions, and online scorecards to submit their reviews using both scoring systems.

All pilot study proposals were first screened for appropriateness based on proposal aims and overall responsiveness to ACTRI DISC request for application priorities (e.g., clear focus on a health condition prioritized in the San Diego Community Health Assessment [7]). After screening, proposals were randomly assigned and independently scored by two reviewers. Reviewers were invited to report any conflicts of interest with their assignments prior to rating. Following independent proposal scoring, reviewers participated in a group meeting to share their experiences using both scoring systems and to finalize the scoring decisions. A follow-up survey that included open-ended response options for qualitative feedback was sent to the reviewers to reflect on their individual experiences using each scoring system. This process was repeated for two review cycles, in 2020 and 2021.

## Results

Ten proposals (7 from the 2020 cycle and 3 from the 2021 cycle) were received and reviewed as part of this study. Table 1 displays the descriptive statistics for the NIH and INSPECT scores averaged across reviewers. Average NIH scores ranged from 2.5 to 6.4 (out of 9), and average INSPECT scores ranged from 10.5 to 23.5 (out of 30). The mean NIH score was 4.1 (SD = 1.2). This table also presents a comparison of the descriptive statistics between the current INSPECT study and the original INSPECT study [5]. In the original INSPECT study, the mean INSPECT score was 9.2 (SD = 7.5) across 30 proposals compared to the current study in which the mean INSPECT score was 17.9 (SD = 4.5).

**Table 1:** Summary scores for NIH and INSPECT ratings in the current study and INSPECT scores from Crable et al. [5]

	**NIH from the current study (*n* = 10 proposals received in 2020, 2021)**	**INSPECT from the current study (*n* = 10 proposals received in 2020, 2021)**	**INSPECT from Crable et al. **[5]** (*n* = 30 proposals, received in 2016, 2017)**
Mean	4.1	17.9	9.2
Median	3.8	19.8	7
Mode	3.8	–	6
Range	2.5–6.4	10.5–23.5	0–26
Standard deviation	1.2	4.5	7.5

NIH scores range from 1 to 9 with lower scores indicating more favorable ratings. INSPECT scores range from 0 to 30 with higher scores reflecting more favorable ratings

Table 2 displays the distribution of criterion-specific INSPECT scores assigned by reviewers in the current study. Proposals received the highest scores for the criteria rating “the care or quality gap,” “evidence-based treatment to be implemented,” and “stakeholder priorities, engagement in change.” The lowest scores were for the criteria rating “feasibility of proposed research design and methods,” “setting’s readiness to adopt new services/treatment/program,” and “policy/funding environment; leverage or support for sustaining change.”

**Table 2:** Criterion-specific INSPECT rating frequencies for the DISC ACTRI pilot review of *n* = 10 proposals

INSPECT criterion	Rating scale				Total average, M (SD)
	**0**	**1**	**2**	**3**	
**The care, quality, community gap, or need**	0 (0%)	1 (6%)	9 (50%)	8 (44%)	2.39 (0.61)
**The evidence-based treatment to be implemented**	0 (0%)	4 (22%)	9 (50%)	5 (28%)	2.06 (0.73)
**Conceptual model, theory or framework, and theoretical justification**	0 (0%)	7 (39%)	7 (39%)	4 (22%)	1.83 (0.79)
**Stakeholder priorities and engagement in change**	1 (6%)	4 (22%)	9 (50%)	4 (22%)	1.89 (0.83)
**Settings readiness to adopt new services/treatment/programs**	2 (11%)	10 (56%)	5 (28%)	1 (6%)	1.28 (0.75)
**D&I strategy/process**	3 (17%)	3 (17%)	9 (50%)	3 (17%)	1.67 (0.97)
**Team experience with setting, treatment, and D&I process**	0 (0%)	3 (17%)	12 (67%)	3 (17%)	2.00 (0.59)
**Feasibility of proposed research design and methods**	3 (17%)	6 (33%)	9 (50%)	0 (0%)	1.33 (0.77)
**Measurement and analysis**	2 (11%)	8 (44%)	3 (17%)	5 (28%)	1.61 (1.04)
**Policy/funding environment and leverage or support for sustaining change**	3 (17%)	7 (39%)	5 (28%)	3 (17%)	1.44 (0.98)

We observed a statistically significant inverse correlation (*r* =  − 0.78, *p* < 0.01) between the average proposal ratings using the NIH criteria and the average proposal ratings using INSPECT. This is consistent with the original INSPECT study that also observed a moderate inverse correlation (*r* =  − 0.62, *p* < 0.01) [5].

Finally, we elicited post-review feedback from the five reviewers of our 10 DIS pilot study proposals. Their feedback was categorized into (1) reflections about the training and review experience and (2) recommendations for refining INSPECT and the review process. A general reflection was that the NIH criteria had a broad scientific purview and were better suited to evaluate effectiveness-focused (e.g., hybrid study designs) and pre-implementation proposals that did not aim to test the specific implementation or dissemination strategies. One reviewer commented, “research proposing novel D&I methods may be better suited for NIH criteria, while INSPECT criteria may be better suited for applied D&I research.” Reviewers reflected that INSPECT was perceived as a more objective rating system and better suited to rate the quality of integrating DIS considerations and to assess the potential for generalizability, real-world feasibility, and impact. Overall, the reviewers reflected that participating in the training and application of INSPECT review criteria were helpful experiences that strengthened their confidence in DIS grant writing. For example, one reviewer commented, “I really liked having a reviewer training meeting. Previously, I had not been part of a proposal review process that included a training. The training gave me time to get familiar with reviewer requirements and talk through any questions I had ahead of time.”

With regard to the refinements to INSPECT and the review process, reviewers recommended that more explicit guidance be offered for using the INSPECT criteria when assessing pre-implementation, formative proposals. For example, for the fifth criterion, “setting readiness,” instead of rating based on an adequate “description of the setting’s interest in the proposed intervention,” this could be modified for pre-implementation research to read, “description of rationale or interest for investigating the setting or the proposed innovation.” Reviewers also recommended that reviewers be encouraged to provide additional written commentary to contextualize their INSPECT ratings. Finally, reviewers felt that the INSPECT requirements for criterion 2, the “evidence-based practice to be implemented,” overlapped with the requirements for criterion 10 “policy/funding’ environment” because both require proposals to include language about how the focal practice/intervention might impact the study setting. Another example of reviewer feedback related to criterion 1 concerning subtle language differences between scores 0 and 1. To obtain a score of 0, the “proposed D&I study/project *is not* linked to a setting” while a score of 1 replaces “is not” with “does not explicitly.” Reviewers preferred greater clarity between these descriptions to yield more consistent scoring.

## Discussion

This study expanded the application and identified new opportunities of the INSPECT proposal review criteria developed by Crable and colleagues [5]. INSPECT scores assigned by reviewers of the 10 proposals of our ACTRI DISC were notably higher (an average of 9 points), compared to the original INSPECT study conducted only 4 years prior. Although it is not possible to assert causative effects underlying the difference in INSPECT ratings, there has been a proliferation of DIS training and educational offerings, many free and online, over the past few years [8]. Our DIS Center has contributed to these free and virtual events including a pre-proposal webinar that offers a primer on DIS terminology, methods, and key ingredients to grant writing and a recent 2-day online workshop focused on how to obtain funding for DIS research [9]. As of the date of this publication, our ACTRI DIS Center has amassed over 500 members, with approximately 13% endorsing that they have advanced D&I skills (Viglione C, Rabin B, Fang O, Scheckter L, Aarons GA, Brookman-Frazee L, Stadnick NA: Evaluation of productivity and impact of an academic dissemination and implementation science capacity building program, submitted). These concerted efforts to offer accessible DIS training may have positively impacted the higher quality of DIS pilot study proposals observed in our study.

Consistent with Crable and colleagues [5] was the inverse correlation between the average NIH ratings and the average INSPECT ratings. This consistent finding affirms the validity of the INSPECT criteria in evaluating proposal quality: proposals that scored well under NIH also scored well under INSPECT; proposals that scored poorly under NIH also scored poorly under INSPECT.

To meaningfully use the INSPECT criteria for the ACTRI DISC pilot proposals, we needed to adapt the original INSPECT published by Crable et al. [5] in eight specific ways. These adaptations were primarily focused on broadening language to apply to diverse D&I proposals more flexibly across the translational spectrum. Noteworthy language revisions included changing “implementation and improvement science” to “dissemination and implementation science” throughout; incorporating “community gap or need” in addition to “quality or care gap”; removing the focus on safety net settings to instead invite research conducted across broad “clinic, community setting, healthcare system, etc.”; changing “theory” to “conceptual model, theory or framework”; and replacing “treatment” with “intervention.”

In addition to the language adaptations, we instituted two procedural adaptations. The first was a specification that letters of support to indicate a setting’s readiness to adopt a change of services/treatments/programs were optional. This adaptation was important because the pilot proposal was designed to be a brief application and in primary service of jumpstarting a line of implementation research that could be built upon in a subsequent larger-scale project. In addition, the ACTRI DISC leadership that developed the RFA was mindful that the timeline required to respectfully request and the craft letters of support do not always align with the timeline for grant submissions. The second procedural adaptation was including an open-text response area for reviewers to describe their justification for each numerical INSPECT criterion rating. This adaptation was instituted to encourage thoughtful rating assignments by reviewers, facilitate generating engaged discussions in the proposal review committee, support the proposal review learning process, and align with the rating process in the NIH scoring procedure in which each criterion rating needs to be justified by a written description of proposal strengths and weaknesses.

These language and procedural adaptations were contextually important to facilitate the ease and applicability of INSPECT for the current study’s pilot proposals. Although these adaptations were specific to our ACTRI DISC’s local context and responsive to a relatively small number of pilot proposals, our described adaptations may be more generalizable to other academic research and community settings than the original INSPECT criteria. From our center’s experience, we recommend that other institutions or programs that endeavor to use INSPECT similarly review each criterion to ensure that the criteria can be meaningfully applied to the DIS proposals invited and received. Our experience suggests that even after adapting INSPECT to local funding priorities, the criteria maintain their validity for evaluating implementation science proposal quality.

INSPECT ratings from the current study highlighted the potential areas for targeted training for those applying for DIS pilot proposals and for the reviewers who had varying levels of experience reviewing NIH proposals. These coalesced around the criteria focused on setting readiness and feasibility of the proposed design and methods. For training, it might be important to focus on how to strategically build meaningful relationships to promote readiness in a given context, as well as selecting pragmatic methods that balance rigor and feasibility, particularly for the scope of a pilot project.

## Conclusions

Overall, the findings of this study confirmed complementarity in using both NIH and INSPECT scoring criteria in our pilot study application reviews. Our reviewers reflected that the NIH review criteria might be better suited for formative research whereas the INSPECT scoring criteria might be better suited for proposals that have well-defined dissemination or implementation strategies and targeted implementation outcomes. Reviewers identified several opportunities for refining the INSPECT criteria and our grant review process that integrates the NIH and INSPECT ratings. These refinements primarily coalesced around greater clarity in applying the INSPECT criteria for pre-implementation research proposals and explicit guidance on differentiating the INSPECT criteria from the overlapping rating criteria.

As DIS research continues to gain traction, a complementary focus on the proposal review criteria used to evaluate DIS research is warranted. One context where this might have specific implications is for NIH Clinical and Translational Science Award programs that will be newly required to engage in DIS activities per the latest program announcement [10]. Additional research and practical application of INSPECT are recommended to facilitate rigorous DIS proposal reviews in the ultimate service of high-impact DIS research.

## Supplementary Information

**Additional file 1.** 

**Additional file 2.** Reporting of Organizational Case Studies Checklist.

## Data Availability

The datasets and reviewer training materials used for the current study are available from the corresponding author upon request.
