# Supplementary material for: Enhancing review criteria for dissemination and implementation science grants
Source: Implement Sci Commun. 2023 Feb 21;4:17. doi: 10.1186/s43058-023-00399-2 (PMC9945623; doi:10.1186/s43058-023-00399-2)
Supplement: Supplementary file 1 — Additional file 1. [file 43058_2023_399_MOESM1_ESM.docx]

| **Additional File. INSPECT Criteria Adapted for UC San Diego ACTRI Dissemination and Implementation Science Center Pilot Grants**  *Instructions for reviewers: Please assign a rating from 0-3 for each of the 10 criteria in the table. Guidance on scores for each criterion are included below.* | | | | |
| --- | --- | --- | --- | --- |
| Criteria | **0** | **1** | **2** | **3** |
| 1. The care, quality, community gap or need | • No care/quality/community gap or need is defined; an issue may be presented, but it is not described as a gap in quality or care | • Unclearly defined care/quality/community gap or need is poorly supported with inappropriate/inadequate/irrelevant local setting data (i.e., evidence of chart review or other preliminary data) or citations from the literature | • Defined care/quality/community gap or need is supported by either local setting data (i.e., evidence of chart review or other preliminary data) or citations from the literature | • Clearly defined care/ quality/community gap or need is supported by local setting data (i.e., evidence of chart review or other preliminary data) and appropriate citations from the literature |
|  | • No information or lack of clarity in the information cited about the potential for improvement or the impact of the proposed D&I study/project to address the stated care/quality/community gap or need | • Insufficient information about the potential for improvement or the impact of the proposed D&I study/project to address the stated care/quality/community gap or need | • Adequate information about the potential for improvement, but would benefit from further specification | • Explicit, well thought out description of the potential for improvement to address the stated care/quality/community gap or need |
|  | • Proposed D&I study/project is not linked to a setting (e.g., clinic, community setting, healthcare system, etc.) | • Proposed D&I study/project does not explicitly link to a setting (e.g., clinic, community setting, healthcare system, etc.) | • Proposed D&I study/project links to a setting (e.g., clinic, community setting, healthcare system, etc.) but may need further clarification | • Proposed D&I study/project is clearly linked to a setting (e.g., clinic, community setting, healthcare system, etc.) |
| 1. The evidence-based treatment to be implemented | • No evidence-based or evidence-informed intervention is identified or the intervention justification/background is based on zero/inappropriate/inadequate citations from literature | • Some literature is cited to provide evidence of limited prior efficacy studies concerning the planned intervention, meeting “evidence-informed” rather than “evidence-based” criteria | • Sufficient literature is cited demonstrating evidence of prior efficacy studies using the intervention to meet either “evidence-informed” or “evidence-based” criteria | • Clearly discusses evidence of prior efficacy studies concerning the planned intervention, meeting “evidence-based” rather than “evidence-informed” criteria |
|  | • Lack of clarity about why the intervention was chosen for the study setting, population, or to address the above stated care/quality/community gap or need | • Limited justification about why the intervention was chosen for the study setting, population, or to address the above stated care/quality/community gap or need and/or justification is based on desire to document efficacy of the proposed evidence-informed practice | • If the intervention is “evidence-informed,” the innovative use of said intervention in the study setting is compelling enough to consider, or there is appropriate justification about why the evidence-based intervention was chosen for the study setting, population, or to address the above stated care/quality/community gap or need, and the goal is not based on developing efficacy of the said evidence-informed practice | • Explicit, well thought-out rationale for implementing the intervention in the selected setting, population, or to address the above stated care/quality/community gap or need including the potential effect it will have on that setting, population, or in addressing the above stated care/quality/community gap or need |
|  | • Unclear what effect the intervention will have on the selected setting, population, or on the above stated care/quality/community gap or need | • Insufficient information describing what effect the intervention will have on the selected setting, population, or on the above stated care/quality/community gap or need | • Adequate information describing what effect the intervention will have on the selected setting, population, or on the above stated care/quality/community gap or need, but may need further clarification |  |
| 1. Conceptual model, theory or framework and theoretical justification | • No conceptual model, framework, or other theoretical grounding is discussed | • A conceptual model, framework, or other theoretical grounding is mentioned, but not linked to the study objectives, hypotheses, and measures | • A conceptual model, framework, or other theoretical grounding is linked in some capacity to the study objectives, hypotheses, and measures, but may need additional clarification | • A D&I conceptual model or framework is clearly described, with the operationalization of theoretical constructs explicitly described within the proposed setting and population |
|  | • Some conceptual model is cited but its basis and constructs are irrelevant to study objectives and/or the study setting | • The chosen conceptual model, framework, or other theoretical grounding may be appropriate for the proposed D&I study/project, but the rationale is not clearly supported with citations from the literature | • The chosen conceptual model, framework, or other theoretical grounding is appropriate for the proposed D&I study/project as evidenced by a well-defined rationale with adequate citations from the literature, but would still benefit from further specificity | • The D&I and/or improvement science-specific conceptual model or framework is used to frame the proposed study/project in all aspects including the study questions, aims/objectives, hypotheses, process, and outcome measures |
|  |  |  |  | • Some discussion may refer and describe how study findings would build upon or otherwise contribute to theory or the larger D&I fields |
| 1. Stakeholder priorities, engagement in change | • Zero or extremely limited description of who the stakeholders (e.g., consumers/service recipients, providers, leaders, policymakers) are or what their preferences and priorities are around the proposed intervention | • Limited description of who the stakeholders are, with some key players missing from consideration | • Sufficient description of who all of the identifiable stakeholders are | • Comprehensive description of who all of the identifiable stakeholders are |
|  | • No evidence of stakeholder analysis planning, or basic information gathering is discussed in relation to how the applicant developed the D&I strategies | • Limited understanding of stakeholder priorities and concerns related to the intervention is demonstrated by easily identified potential issues that are not discussed in the application, or no evidence of stakeholder analysis planning is discussed | • Clear understanding of stakeholder concerns related to the intervention as evidenced by a stakeholder analysis plan that describes how the applicant will collect at least some information on stakeholders interests, interrelations, influences, preferences, or/and priorities | • Clear understanding of stakeholder concerns related to the intervention as evidenced by a stakeholder analysis plan that describes how the applicant will collect comprehensive information on stakeholders interests, interrelations, influences, preferences, and priorities |
|  |  | • Zero or very limited mention of involving stakeholders in the conceptual design of the intervention, and/or consideration of the D&I strategies, process, or outcomes | • Somewhat unclear description of how stakeholders were involved in the conceptual design of the intervention, and/or consideration of the D&I strategies, process, or outcomes | • Detailed description of how stakeholders were involved in the conceptual design of the intervention and in considering the D&I strategies, process, and outcomes |
|  |  | • No clear agreement or collaboration between the stakeholders and the applicant is explained | • Some type of agreement or collaboration between the stakeholders and the applicant is explained but supporting evidence is limited | • An explicit agreement (such as a memorandum of understanding) or evidence of collaboration between the stakeholders and the applicant that is explained with relevance to the proposed study process and how findings will be communicated |
| 1. Settings readiness to adopt new services/treatment/programs | • Zero or very limited rationale/interest for implementing the proposed intervention in the identified setting is discussed | • Some description of the setting’s interest in the proposed intervention | • Clearly describes the setting’s interest and rationale for the proposed intervention | • Explicitly describes preliminary data on the assessed organizational and political capacity and readiness for implementation (assessment completed prior to current D&I study/project |
|  | • No information on the study setting’s capacity or readiness for implementation | • Incomplete or unclear description of how the setting will be assessed for capacity and/or readiness for implementation including which methods and tools will be used, or there is a limited description of organizational/political culture and potential contextual barriers or facilitators | • Clearly describes how the setting will be assessed for capacity and readiness for implementation including which methods, scales, or other tools will be used | • Preliminary capacity and readiness assessments were completed using a scale with established validity and reliability, or a scale that has undergone some validity and reliability testing |
|  | • No information on how those in the study setting who are opposed to change will be involved with or have their concerns addressed by study processes or components | • May include a brief discussion on how those opposed to change in the study setting will be involved with or have their concerns addressed by study processes or components | • Thoroughly describes the potential influence of organizational/political culture, and potential contextual barriers or facilitators | • May include strategies for how those opposed to change in the study setting will be involved with or have their concerns addressed by study processes or components |
|  |  | • May not include evidence of support (e.g., letters) from the study setting that address how the proposed study aligns with the organization’s priorities/policies | • May include strategies for how those opposed to change in the study setting will be involved with or have their concerns addressed by study processes or components | • Evidence of support (e.g., letters) from the study setting that address how the proposed study aligns with the organization’s priorities/policies. *Note: letters of support are not required as part of the DISC pilot RFA but can be considered here if submitted.* |
|  |  |  | • May not include evidence of support (e.g., letters) from the study setting that address how the proposed study aligns with the organization’s priorities/policies |  |
| 1. D&I strategy/process | • No D&I strategies are identified | • D&I strategies are not clearly distinguished from the intervention | • D&I strategies are clearly distinguished from the intervention | • Explicitly describes and theoretically justifies the D&I strategies |
|  | • The clinical intervention may be incorrectly described a D&I strategy | • Unclear D&I strategies are not theoretically justified and/or do not match with the stated aims/setting/outcome measures of the proposed study | • Some theoretical justification of the D&I strategies | • Explicitly describes how D&I strategies link to the stated aims/setting/outcome measures of the proposed study |
|  |  | • Limited description linking the D&I strategies to the stated aims/setting/outcome measures of the proposed study with no plan for how strategies will be observed or tested | • Clearly describes how D&I strategies link to the stated aims/setting/outcome measures of the proposed study | • Explicitly describes how D&I strategies will be observed or empirically tested |
|  |  | • D&I strategies may be unrealistic given the pilot timeline and/or budget constraints | • More description is needed to clearly understand how D&I strategies will be observed or empirically tested | • D&I strategies are feasible given the pilot study timeline and budget constraints |
|  |  |  | • D&I strategies are mostly feasible given the pilot study timeline and budget constraints |  |
| 1. Team experience with setting, treatment, and D&I process | • Only the principal investigator’s skills are described | • It is unclear how the team experience relates to the study setting, population, intervention, and/or processes | • Team description, biographical sketches, resumes/CVs depict a multidisciplinary skillset relevant to the proposed study setting, population, intervention, processes, and other needs | • Clearly describes how team experience relates to the study setting, population, intervention, and processes |
|  | • No additional information, biographical sketches, resumes/CVs are provided beyond the principal investigator | • Staffing plan may not facilitate successful study completion | • Staffing plan facilitates successful study completion, with some potential shortage | • Team description, biographical sketches, resumes/CVs depict a multidisciplinary skillset relevant to the proposed study setting, population, intervention, processes, and other needs |
|  |  | • Team experience is uniform and does not offer multidisciplinary skills or perspective to the proposed study | • No description of the research environment strengths including resources and/or infrastructure | • Staffing plan facilitates successful study completion without necessitating additional support |
| 1. Feasibility of proposed research design and methods | • The proposed study includes methods, interventions, and other components that are beyond the scope of a pilot study and/or inappropriate for a pilot study | • The proposed study includes methods, interventions, and other components that may be challenging to accomplish | • The proposed study includes appropriate methods, interventions, and other components that are likely achievable as a pilot study | • The proposed study includes appropriate methods, interventions, and other components that are achievable as a pilot study and are justified against potential alternatives |
|  | • A budget and/or timeline are not included or are unrealistic | • The budget and/or timeline are not included or unrealistic | • The budget and/or timeline may need some revising | • The budget and timeline are appropriate |
|  | • Potential barriers to dissemination and/or implementation are not described or are insurmountable | • Potential barriers to dissemination and/or implementation are not clearly described or are insurmountable | • Potential barriers to dissemination and/or implementation are clearly described but may lack clear description of how those barriers will be overcome | • Potential barriers to dissemination and/or implementation are clearly identified with potential plans to overcome those barriers |
| 1. Measurement and analysis section | • Outcomes described are not D&I science-related | • Outcomes described are D&I science related | • Outcomes described are D&I science related | • Outcomes described are D&I science-related |
|  | • Outcomes are not linked to the proposed study aims | • Outcomes are unclearly linked to the proposed study aims | • Outcomes are clearly linked to the proposed study aims | • Outcomes are clearly linked to the proposed study aims |
|  | • The unit of analysis is inappropriate for the proposed study | • The unit of analysis is appropriate for the proposed study | • The unit of analysis is appropriate for the proposed study | • The unit of analysis is appropriate for the proposed study |
|  | - No information on the quality (i.e., psychometric strength and pragmatic characteristics such as length, cultural fit, etc.) of the proposed measures | - Some information on the quality of the proposed measures (e.g., internal consistency reported about a proposed measure or some pragmatic characteristic). | - Adequate information about the psychometric and/or pragmatic qualities of the proposed measures. | - Comprehensive reporting of both the psychometric and pragmatic qualities of the proposed measures. |
| 1. Policy/funding environment; leverage of support for sustaining change | • No acknowledgement of the internal/external policy trends and/or funding environment for the proposed study is included | • The internal/external policy trends and/or funding environment are discussed but additional clarification is needed | • The internal/external policy trends and/or funding environment are clearly described | • The internal/external policy trends and/or funding environment are clearly described |
|  | • Zero or limited discussion of the potential impact of the intervention and/or D&I strategy is included | • The potential impact of the intervention and/or D&I strategy is not linked to the policy and/or funding context and may not be relevant to the proposed setting, population, care/quality/community gap or need | • The potential impact of the intervention is linked to relevant policies and funding issues associated with the proposed setting, population, care/quality/community gap or need but may need further explanation | • Potential impact of the intervention is explicitly linked to relevant policies and funding issues associated with the proposed setting, population, care/quality/community gap or need |
|  | • Zero or limited discussion of disseminating study findings is included | • The dissemination plan for study findings does not clearly indicate a contribution will be made to the broader policy level, practice, and community settings | • The dissemination plan for study findings indicates a contribution will be made to the broader policy level, practice, and community settings, but what contribution and how it will be achieved is unclear | • The dissemination plan for study findings indicates what and how a contribution will be made to the broader policy level, practice, and community settings |

*Note*. ^1^Adapted from Crable EL, Biancarelli D, Walkey AJ, Allen CG, Proctor EK, Drainoni ML. Standardizing an approach to the evaluation of implementation science proposals. Implementation Sci. 2018 Dec;13(1):71. ^2^ For ease of reading, only the final adapted criteria used for the ACTRI DISC pilot proposal reviews is shown.
