# Supplementary material for: Enhancing review criteria for dissemination and implementation science grants
Source: Implement Sci Commun. 2023 Feb 21;4:17. doi: 10.1186/s43058-023-00399-2 (PMC9945623; doi:10.1186/s43058-023-00399-2)
Supplement: Supplementary file 2 — Additional file 2. Reporting of Organizational Case Studies Checklist. [file 43058_2023_399_MOESM2_ESM.docx]

Supplemental File. Reporting of Organizational Case Studies Checklist

| **Reporting item** | **Page number on which item was reported** | **Page number of justification for not reporting** |
| --- | --- | --- |
| **Describing the design** | | |
| 1. Define the research as a case study | 5 |  |
| 2. State the broad aims of the study | 5 |  |
| 3. State the research question(s)/hypotheses | N/A because this was a descriptive, exploratory study without a priori hypotheses or research questions. |  |
| 4. Identify the specific case(s) and justify the selection | 5, 7 |  |
| **Describing the data collection** | | |
| 5. Describe how data were collected | 5-7 |  |
| 6. Describe the sources of evidence used | 5-7 |  |
| 7. Describe any ethical considerations and obtainment of relevant approvals, access and permissions | 16 |  |
| **Describing the data analysis** | | |
| 8. Describe the analysis methods | 7-10 |  |
| **Interpreting the results** | | |
| 9. Describe any inherent shortcomings in the design and analysis and how these might have influenced the findings | 11-12 |  |
| 10. Consider the appropriateness of methods used for the question and subject matter and why it was that qualitative methods were appropriate | 5-7 |  |
| 11. Discuss the data analysis | 10-12 |  |
| 12. Ensure that the assertions are sound, neither over- nor under-interpreting the data | 10-12 |  |
| 13. State any caveats about the study | 10-12 |  |
